# Biochar-amended potting medium reduces the susceptibility of rice to root-knot nematode infections

**DOI:** 10.1186/s12870-015-0654-7

**Published:** 2015-11-04

**Authors:** Wen-kun Huang, Hong-li Ji, Godelieve Gheysen, Jane Debode, Tina Kyndt

**Affiliations:** Department of Molecular Biotechnology, Ghent University, Coupure Links 653, B-9000 Ghent, Belgium; State Key Laboratory for Biology of Plant Diseases and Insect Pests, Institute of Plant Protection, Chinese Academy of Agricultural Sciences, 100193 Beijing, P. R. China; Institute of Plant Protection, Sichuan Academy of Agricultural Sciences, Jingjusi Road 20, 610066 Chengdu, P. R. China; Plant Sciences Unit – Plant Protection, Institute for Agricultural and Fisheries Research (ILVO), Burg. van Gansberghelaan 96, 9820 Merelbeke, Belgium

**Keywords:** Biochar, *Meloidogyne graminicola*, Hydrogen peroxide, Lignin, Callose

## Abstract

**Background:**

Biochar is a solid coproduct of biomass pyrolysis, and soil amended with biochar has been shown to enhance the productivity of various crops and induce systemic plant resistance to fungal pathogens. The aim of this study was to explore the ability of wood biochar to induce resistance to the root-knot nematode (RKN) *Meloidogyne graminicola* in rice (*Oryza sativa* cv. Nipponbare) and examine its histochemical and molecular impact on plant defense mechanisms.

**Results:**

A 1.2 % concentration of biochar added to the potting medium of rice was found to be the most effective at reducing nematode development in rice roots, whereas direct toxic effects of biochar exudates on nematode viability, infectivity or development were not observed. The increased plant resistance was associated with biochar-primed H_2_O_2_ accumulation as well as with the transcriptional enhancement of genes involved in the ethylene (ET) signaling pathway. The increased susceptibility of the *Ein2b*-RNAi line, which is deficient in ET signaling, further confirmed that biochar-induced priming acts at least partly through ET signaling.

**Conclusion:**

These results suggest that biochar amendments protect rice plants challenged by nematodes. This priming effect partially depends on the ET signaling pathway and enhanced H_2_O_2_ accumulation.

## Background

Rice is one of the most frequently consumed cereal foods in the world. Based on current forecasts by the Food and Agriculture Organization of the United Nations (FAO), world rice production in 2015 will reach ca. 500 million tons [1]. However, the number of soilborne pathogens (including nematodes) is increasing worldwide because cultivation practices have been altered to use less water, and these pathogens present a potential threat to rice production [2]. Estimates of the annual yield losses of rice as a result of damage by plant-parasitic nematodes range from 10 to 25 % worldwide [3]. One of the most damaging nematodes to rice is the root-knot nematode (RKN) *Meloidogyne graminicola* (Mg), which causes the formation of galls on the rice roots. After penetrating the root elongation zone and migrating intercellularly towards the root tip, RKNs enter the vascular cylinder, where they puncture the cell wall with their stylet and inject secretions from their pharyngeal glands into the plant cell to induce a permanent feeding site known as giant cells [4, 5]. In intensive cropping systems, RKNs have been managed for decades with chemical nematicides (e.g., temic, furadan and fenamiphos). However, the potential negative impacts of these chemicals to the environment and humans have led to a ban or restricted use of most chemical nematicides. With increased pressure on growers to reduce nematicide usage and without effective alternatives, there is increasing interest in induced resistance (IR) or priming as new management tool for this destructive pathogen.

Priming is a physiological state of enhanced defensive capacity elicited by special stimuli, in which the innate defenses of the plants are potentiated for rapid activation upon subsequent challenge from fungi, bacteria, viruses, or nematodes [6]. In general, two major pathways that lead to enhanced defense in plants have been described, and they are differentiated by the nature of the elicitors and regulatory pathways [7]. Systemic acquired resistance (SAR) is associated with the production of pathogenesis-related (PR) proteins and mediated by a salicylic acid (SA)-dependent process and it usually starts with a hypersensitive reaction that leads to local necrosis. Induced systemic resistance (ISR) is triggered by several mechanisms, such as by plant growth-promoting rhizobacteria (PGPR) and fungi (PGPF), and mediated by a signaling pathway in which the phytohormones ethylene (ET) and jasmonic acid (JA) play key roles [6, 8]. Nahar et al. [9] studied the JA/ET pathways and found that supplying ethephon (a source of ET) or methyl jasmonate to rice shoots induced a strong systemic defense response in the roots against *M. graminicola*. Confirming the importance of JA in plant defense against nematodes, the foliar application of JA was also found to induce systemic defense against RKNs in tomatoes [10]. However, Bhattarai et al. [11] found that JA signaling through coronatine-insensitive 1 (COI1) is required for the susceptibility of tomatoes to RKNs.

The role of ET in root defense against nematodes is also controversial. Nahar et al. [9] reported that ET activation of root defense against RKNs in rice is based on intact JA biosynthesis. Confirming the role of ET in root defense, Fudali et al. [12] showed that ET-overproducing Arabidopsis plants are less attractive to RKNs. However, ET might have a positive effect on root gall development, which was observed by Glazer et al. [13] in tomatoes. Overall, the roles of plant hormone pathways in plant defenses against nematodes might vary depending on the host species and may change over the course of the infection process. These hormone pathways are also not a prerequisite for induced defenses against nematodes, which was shown by Ji et al. [14], who found that the non-protein amino acid beta aminobutyric acid (BABA) can induce defense against RKNs in rice independent of the JA and ET pathways, but rather acts through activation of lignin and callose production.

One of the potential priming agents for induced plant defenses that is currently receiving significant attention is biochar, which is a high-carbon material produced from the slow pyrolysis of biomass in the absence of air and thus a by-product from the biofuel industry [15]. Certain biochar additions to soil have been shown to significantly improve the soil tilth, nutrient retention and availability to plants, and crop productivity [7, 16, 17]. The observed effects on crops resulting from biochar soil amendments have primarily been explained by improved nutrient retention [18], increased pH and altered soil physical properties [19], improved mycorrhizal fungi colonization [20] and altered soil biological community composition and abundance [18, 21]. Several studies have demonstrated that soil-applied biochar can induce systemic defenses in many plants against different foliar fungal pathogens. The fungal foliar diseases *Botrytis cinerea* and *Oidiopsis sicula* in tomato and pepper were significantly reduced in biochar-amended potting medium [7]. Two different biochars were found to induce strawberry plant systemic resistance to three foliar fungal pathogens with different infection strategies: necrotrophic (*B. cinerea*), hemi-biotrophic (*Colletotrichum acutatum*), and biotrophic (*Podospharea aphanis*) [22]. Graber et al. [23] presumed that this resistance might result from either low-level stress exerted by phytotoxic compounds contained in the biochar (e.g., ET and propylene glycol) or through larger populations of beneficial microorganisms isolated from the biochar-treated soils, such as the well-known ISR-inducing *Trichoderma* spp. [24]. Recently, Mehari et al. [25] observed that biochar amendment resulted in an approximately 50 % reduction in *B. cinerea* disease severity in most of the tested genotypes of *Solanum lycopersicum*. The systemic resistance of *S. lycopersicum* induced by biochar amendment was shown to be related to stronger and earlier hydrogen peroxide (H_2_O_2_) accumulation and involved JA signaling.

Currently, data are limited on the effect of biochar on plant parasitism by nematodes. The amendment of poultry-litter biochar to the soil generally decreased the number of plant-parasitic nematodes while increasing the amount of free-living nematodes in the soil [26]. Matlack [27] conducted an observational study at the landscape scale and could not detect a significant relationship between nematode populations and charred materials in the soil. In addition, significant effects were not observed on the total nematode abundance when short-term biochar additions were practiced in wheat fields [28]. However, biochar was found to have a high sorption capacity for dichloropropene, a strong anti-nematode fumigant. As a result, biochar-amendment to the soil can increase the required dose of dichloropropene to efficiently control nematodes [29]. These reports have investigated the effects of biochar in the soil on nematode populations and on chemical nematode control measures, whereas the indirect effects through the activation of plant defenses against parasitic nematode infections have not been investigated.

The present study was designed to test whether soil amended with biochar was capable of inducing resistance in rice plants against the RKN *M. graminicola*. After establishing a beneficial effect, the role of defense-related pathways, the generation of H_2_O_2_, and the deposition of callose and lignin were investigated in the treated and infected rice plants. The role of the ET pathway was further investigated using an ET-insensitive line. We found that biochar-induced defenses in rice against *M. graminicola* involves H_2_O_2_ accumulation in the rice roots and is partially dependent on ET signaling.

## Results

### Biochar exudates do not present negative effects on the survival and infectivity of nematodes

**Fig. 1:**
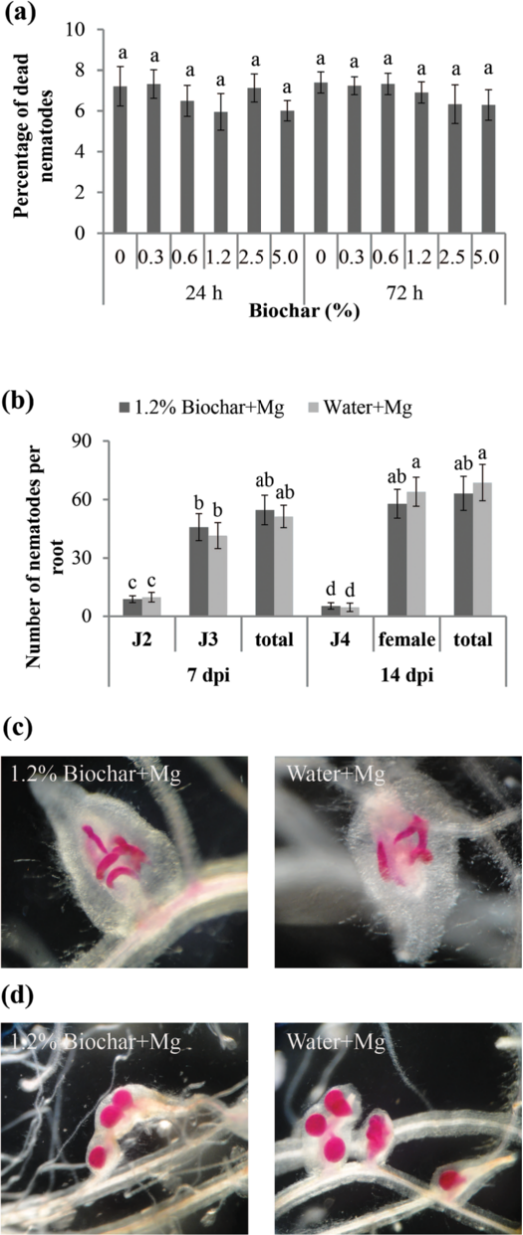
Direct effect of the biochar exudates on the behavior of *M. graminicola* (Mg). **a** Percentage of dead juveniles 24 h and 72 h after incubation in various concentrations of biochar exudates and water. **b** Penetration and development of biochar-incubated and water-incubated *M. graminicola* in rice roots. **c** Biochar-incubated and water-incubated nematodes were inoculated on rice roots and photographed at 7 dpi. **d** Biochar-incubated and water-incubated nematodes were inoculated on rice roots and photographed at 14 dpi. The bars in the different graphs represent the mean ± SE of the data from three independent biological replicates, each containing 6 individual plants. Different letters indicate statistically significant differences (Duncan’s multiple range test at *p* ≤ 0.05). J2: second stage juveniles. J3: third stage juveniles. J4: fourth stage juveniles

To evaluate the nematicidal effect of biochar on *M. graminicola*, the nematodes were incubated in different concentrations of biochar exudates. Significant differences in nematode mortality were not observed between biochar exudates (6.6 ± 0.7 %) and water (7.2 ± 0.6 %) 24 h after initiation of the bioassay at doses ranging from 0.3 to 5 % biochar (Fig. 1a). Similar results were also observed when the nematodes were incubated in biochar exudates for 72 h (Fig. 1a). These data suggest that biochar exudates do not have a direct nematicidal effect on *M. graminicola* at the doses tested.

To verify whether biochar can hamper the infectivity of RKNs, the nematodes treated with biochar exudates or water (as control) were inoculated in rice roots. The nematode penetration and development were recorded at 7 and 14 dpi (Fig. 1b). At 7 dpi, most of the nematodes developed to third-stage juveniles (J3). The mean number of nematodes inside the roots and their development was not different between the biochar-exudate treated and control nematodes (Fig. 1b and c). At 14 dpi, most of the nematodes had developed into adult females. Again, significant differences were not observed in the number of adult females or the total number of nematodes in the biochar exudate-treated and water-treated nematodes (Fig. 1b and d). The ratio of adult females among the biochar exudate-treated nematodes (91.6 ± 7.4 %) was similar to that in the water-treated nematodes (93.2 ± 7.1 %). Overall, our data show that incubation of *M. graminicola* in biochar exudates did not inhibit their penetration or delay their development inside the rice roots.

### Soil amended with biochar reduces the infection of *M. graminicola* in rice without restraining plant growth

**Fig. 2:**
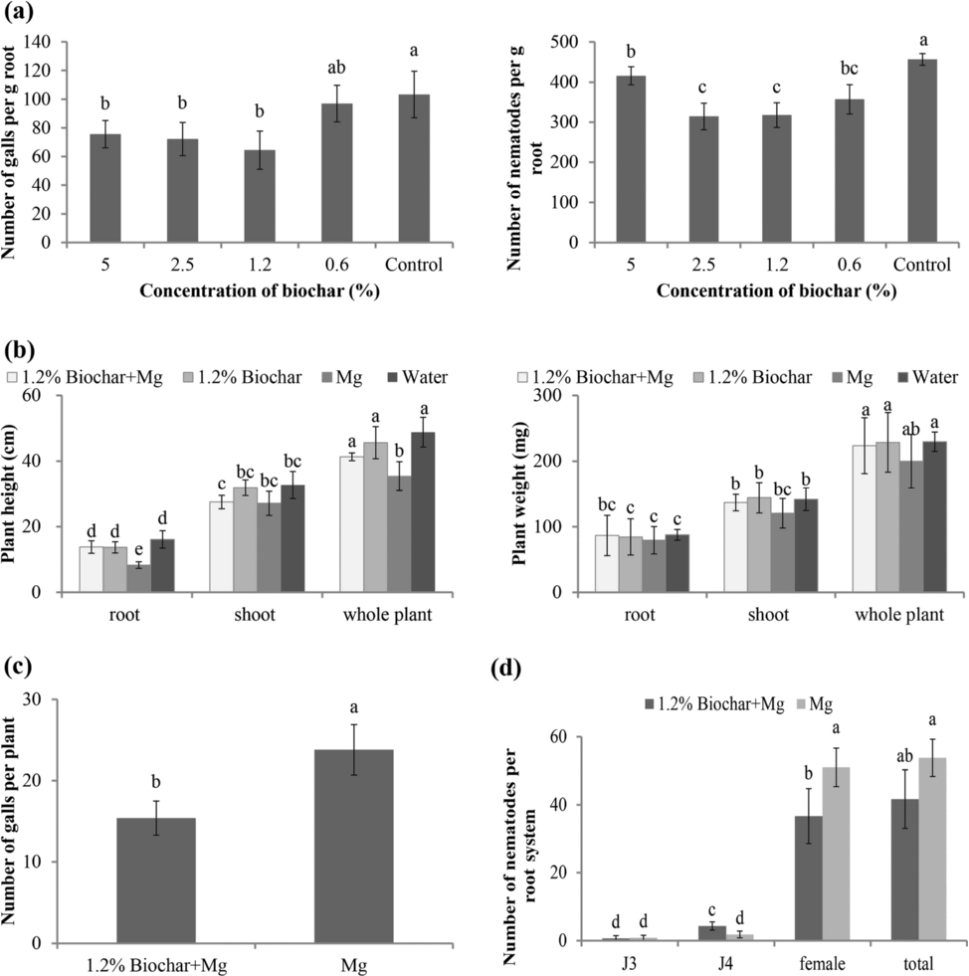
Effects of biochar amendments on plant growth and nematode infectivity in rice roots. **a** Root galls and nematodes per gram root in biochar-amended (different concentrations) and non-amended rice roots were counted at 14 dpi. **b** Plant height and fresh weight were measured at 14 dpi. **c** Root galls per plant on the 1.2 % biochar-amended and non-amended rice roots were counted at 14 dpi. **d** Nematodes per root in different developmental stages in the 1.2 % biochar-amended and non-amended rice roots were counted at 14 dpi. The bars in the different graphs represent the mean ± SE of the data from three independent biological replicates, each containing 6 individual plants. Different letters indicate statistically significant differences (Duncan’s multiple range test at *p* ≤ 0.05)

Recently, relatively low concentrations (1 %) of four biochars prepared from two feedstocks at different pyrolysis temperatures were found to suppress the damping-off of *Rhizoctonia solani* in beans, whereas a higher concentration (3 %) provided ineffective disease protection [30]. Thus, the effect of different biochar doses deserves more attention. To evaluate the potential of biochar as a priming agent, different concentrations of biochar were added to the SAP-substrate, and plant susceptibility was evaluated at 14 dpi. The results revealed that the biochar amendment reduced the number of galls per g of root and the number of nematodes per g of root at all of the tested concentrations (Fig. 2a). However, the best effect was observed at a concentration of 1.2 % biochar in SAP. Therefore, all further experiments were executed with this optimal biochar concentration of 1.2 %.

In subsequent experiments, the growth parameters were assessed by analyzing the length and fresh weight of the roots and shoots of 4-week-old plants (Fig. 2b). When comparing the RKN-infected plants with non-infected plants, slight but significant reductions in the root length and total plant height were observed in the RKN-infected plants. Although 1.2 % biochar alone did not have a significant effect on the analyzed growth parameters, the biochar amendment partially alleviated the negative effects caused by the RKN-infection.

At the optimal concentration of 1.2 %, amendments of biochar significantly reduced the total number of root galls at 14 dpi (Fig. 2c). In addition, the development of nematodes in biochar-amended roots was slightly delayed. The number of adult females in biochar-amended roots was slightly lower than that of non-amended plants, whereas a higher number of fourth-stage juveniles (J4s) were observed in biochar-amended roots compared with that of non-amended plants (Fig. 2d).

**Fig. 3:**
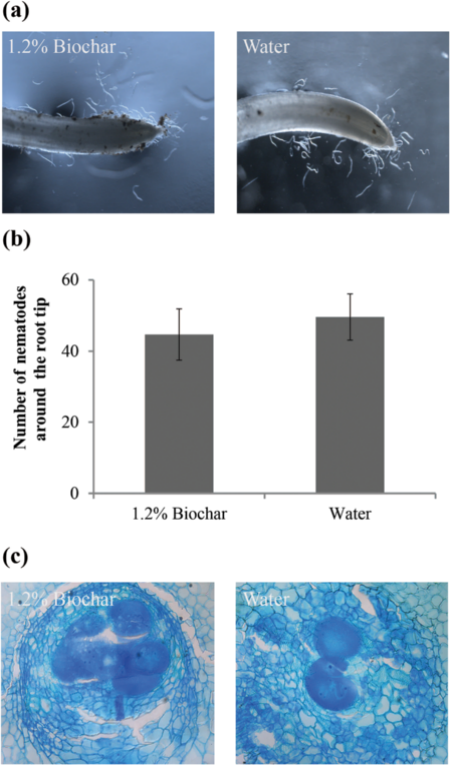
Effect of biochar on the attractiveness of rice roots to *M. graminicola* and microscopic observations of giant cells induced in the root system. **a** Attraction of *M. graminicola* towards the root tips of rice after root drenching with 1.2 % biochar exudates or water were observed under a Leica stereomicroscope with a DFC400 camera. **b** Nematodes in the vicinity of the root elongation zone were counted at 9 hpi. The bars represent the mean ± SE of the data from 6 replicates. No significant differences were found (Duncan’s multiple range test at *p* > 0.05). **c** Sections of giant cells in the biochar-amended root galls and non-amended root galls were stained with toluidine blue and observed at 7 dpi under an Olympus BX 51 microscope with a ColorView III camera. Multiple sections of 10 galls were evaluated and the figure shows one representative section for each treatment

Root exudates often attract nematodes and trigger egg hatching in certain plant-parasitic nematode species [31]. To verify whether biochar impedes the ability of the plant to attract *M. graminicola*, rice roots were drenched with biochar exudates or water 1 d before inoculation. At 9 hpi, approximately 20.2 ± 3.1 % of the nematodes were attracted to the biochar-treated root tips, which was not significantly different from those attracted to the non-amended root tips (23.3 ± 3.2 %) (*p*>0.05) (Fig. 3a, b). This result indicates that the tested biochar exudates do not prevent the attraction of *M. graminicola* to rice.

A microscopic analysis of the nematode feeding sites inside the galls revealed that significant morphological differences did not occur in the giant cells formed in the biochar-amended roots versus the non-amended roots. Most of the giant cells were still enlarged cells with multiple nuclei, dense cytoplasm, and thickened cell walls (Fig. 3c).

These data demonstrate that biochar amendments at a concentration of 1.2 % delay the development of the RKNs but do not change the root attractiveness or the giant cell morphology. However, at this concentration, biochar amendments to the soil can reduce the negative effect of RKNs on plant growth.

### Biochar amendment does not induce callose deposition in root galls

**Fig. 4:**
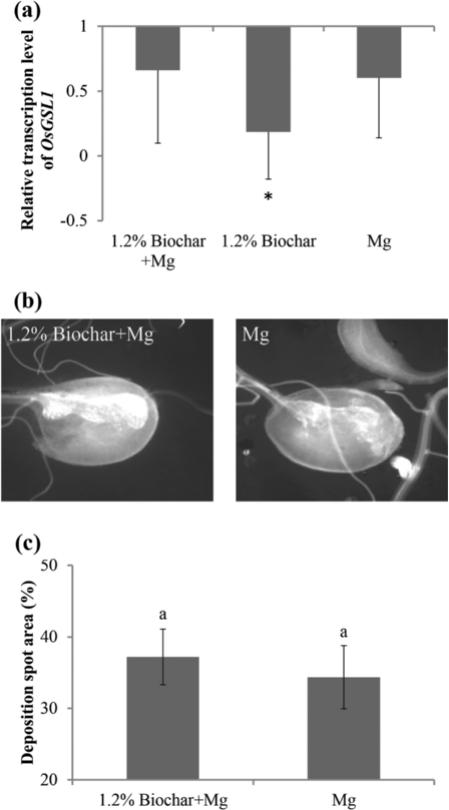
Effect of biochar-amendment to the growth medium on callose biosynthesis in the rice root system. **a** The relative transcript levels of a callose biosynthesis gene (glucan synthase-like gene, *OsGSL1*) at 24 hpi were analyzed using qRT-PCR. The gene expression levels were normalized using three internal reference genes, *OsEXP*, *OsEif5C* and *OsEXPnarsai*. The data are shown as the relative transcript levels normalized to the control roots (expression level in the control set at 1). The bars represent the mean expression level ± SE from two independent biological replicates , each containing a pool of 6 plants. Asterisks indicate significantly different expression levels in comparison with the control roots. **b** Callose deposition in the root galls at 7 dpi was examined under UV light using a Nikon Eclipse Ti-E epifluorescence microscope (excitation 390 nm; emission 460 nm). **c** Quantification of callose deposition was performed using ImageJ software. The data presented are the mean ± SE of two independent experiments, each performed using ten galls. Different letters indicate significant differences (Duncan’s multiple range test at *p* ≤ 0.05)

The addition of BABA to protect rice plants from RKNs was previously shown to be correlated with enhanced glucan synthase-like gene (*OsGSL1*) mRNA levels and callose deposition in the gall tissue [14]. To investigate whether biochar has a similar mode of action, the expression of this callose synthase-encoding gene, *OsGSL1*, was investigated by quantitative reverse transcriptase PCR (qRT-PCR) in biochar-amended and non-amended plants. At 24 hpi, the transcription level of *OsGSL1* was significantly down-regulated in the biochar-amended plants compared with the control plants (Fig. 4a). Significant differences were not observed in inoculated plants, whether biochar-amended or non-amended, although in both cases, a trend towards lower expression of this gene was observed. Confirming these results, the prominence and density of callose spots in biochar-amended galls were similar to those in the non-amended galls at 7 dpi (Fig. 4b and c). These data suggest that biochar amendments do not induce callose deposition after nematode invasion.

### Biochar amendment induces H_2_O_2_ accumulation but not lignification in root tissues

**Fig. 5:**
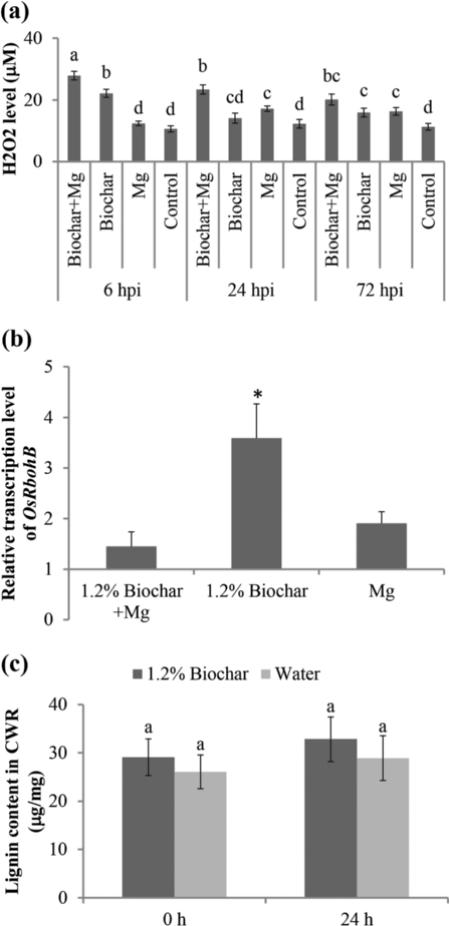
Effect of biochar-amendment to the growth medium on H_2_0_2_ accumulation and lignin levels in the rice roots. **a** H_2_O_2_ content per gram of root was measured upon reaction with KI and detection using a CLARIOstar Microplate Reader at 390 nm. The bars represent the mean ± SE of four replicates, each containing a pool of six roots. Different letters indicate significant differences (Duncan’s Multiple Range Test at *p* ≤ 0.05). **b** Relative transcript levels of the H_2_O_2_ synthesis gene (*OsRbohB*) at 24 hpi were analyzed using qRT-PCR. The gene expression levels were normalized using three internal reference genes, *OsEXP*, *OsEif5C* and *OsEXPnarsai*. The data shown are the relative transcript levels compared with the control roots (expression level set at 1). The bars represent the mean expression level ± SE from two independent biological replicates, each containing a pool of 6 plants. Asterisks indicate significantly different expression levels in comparison with water-treated control roots. (*p* ≤ 0.05). **c** Lignin content in the roots of rice amended with 1.2 % biochar or water was determined using the acetylbromide assay. Root samples were collected before inoculation (0 h) and 24 hpi. The bars represent the mean ± SE of the lignin content of 6 plants. Different letters indicate significant differences (Duncan’s multiple range test at *p* ≤ 0.05)

H_2_O_2_ is an important reactive oxygen species (ROS) and essential to the induction of defense responses in plants. This experiment was conducted to investigate whether biochar is capable of generating ROS for the induction of defense against *M. graminicola*. First, the H_2_O_2_ levels were measured in the plant roots at three different time points, and the results showed that biochar amendments alone led to higher H_2_O_2_ levels in the rice roots (Fig. 5a). Upon *Mg*-inoculation (in non-amended SAP), an increase in the H_2_O_2_ levels was also observed. However, in biochar-amended inoculated plants, the H_2_O_2_ levels increased to significantly higher levels at all of the investigated time points, indicating a priming effect. A quantitative analysis of *OsRbohB*, an NADPH oxidase gene involved in the plant immune response [32], showed that the transcription level of *OsRbohB* was significantly up-regulated in plants that received biochar amendment alone compared with non-amended non-inoculated control plants at 24 hpi (Fig. 5b). However, significant differences were not observed in biochar-amended inoculated plants and non-amended inoculated plants. Most likely, the root knot nematode interferes with the induction of this gene or its induction happens at other time points than those studied here.

The increased production of H_2_O_2_ is known to cause the polymerization of monolignols by peroxidase and subsequent formation of lignin [33]. Lignin confers mechanical strength to plant secondary cell walls, which contributes to basal defenses against plant-parasitic nematodes [34]. Prior to nematode inoculation (0 h), the lignin level in the roots receiving the biochar amendment alone was similar to that in the non-amended roots (Fig. 5c). At 24 hpi, slightly stronger lignification was observed in the biochar-amended inoculated roots, although the difference was not statistically significant. These data indicate that biochar amendments do not strongly promote lignin synthesis.

### Biochar-induced defense in rice against *M. graminicola* is partly mediated by the activation of ET signaling

ET can be produced from the pyrolysis of biomass, although the production of ET varied drastically across different evaluated biochars [35]. To investigate the importance of the ET pathway in biochar-induced resistance against RKNs, the expression levels of genes involved in ET responses (*OsERF70*, *OsERF1, OsEBP89*), ET biosynthesis (*OsACS1*, *OsACO7*) and ET signaling (*OsEIN2*) were analyzed.

**Fig. 6:**
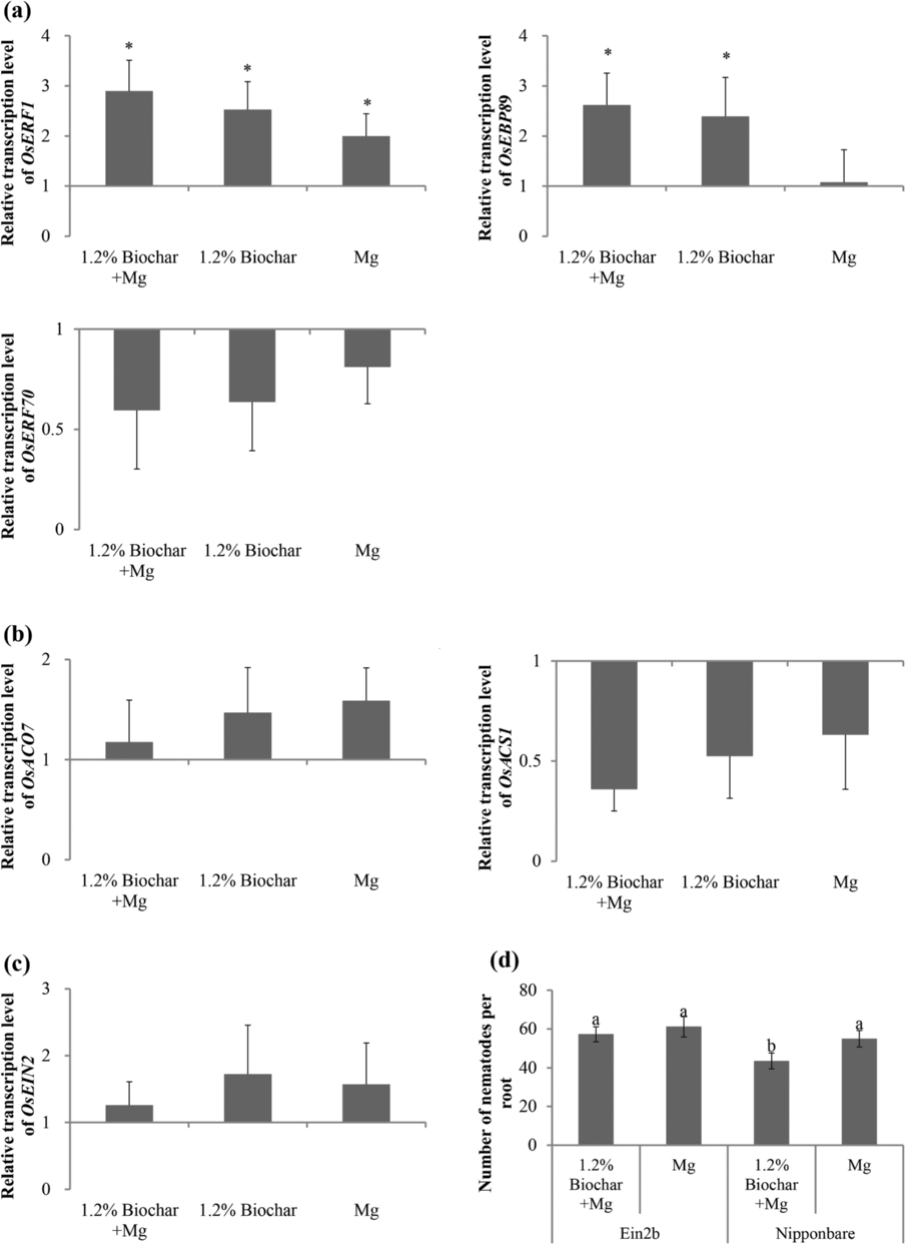
The effect of biochar-amendment in the rice growth medium on the ET-pathway in the rice roots. **a** Relative expression levels of *OsERF1*, *OsEBP89*, *OsERF70*, which are involved in the ethylene response pathway, were analyzed at 24 hpi using qRT-PCR. **b** Relative expression levels of *OsACO7* and *OsACS1*, which are involved in the ethylene biosynthesis pathway, were analyzed at 24 hpi using qRT-PCR. **c** Relative expression levels of *OsEIN2*, which is involved in the ethylene signaling pathway, were analyzed at 24 hpi using qRT-PCR. The gene expression levels were normalized using three internal reference genes, *OsEXP*, *OsEif5C* and *OsEXPnarsai*. The data shown are the relative transcript levels compared with the control roots (expression level set at 1). The bars represent the mean expression level ± SE from two independent biological replicates and three technical replicates, each containing a pool of 6 plants. Asterisks indicate significantly different expression levels (*p* ≤ 0.05). 1.2 % Biochar + Mg, 1.2 % biochar amendment plus *M. graminicola* inoculation; 1.2 % Biochar, 1.2 % biochar amendment alone; Mg, *M. graminicola* inoculation alone; control, non-amended and non-inoculated. **d** Effect of an *Ein2b*-RNAi mutant, which is deficient in ethylene signaling, and the wild type Nipponbare on nematode infection at 14 dpi. The bars represent the mean of the data from three independent biological replicates, each containing 6 plants. Different letters indicate significant differences (Duncan’s multiple range test at *p* ≤ 0.05)

The transcription of the ET response genes *OsERF1* and *OsEBP89* was significantly up-regulated in the biochar-amended plants (Fig. 6a), whereas *OsERF70* was not significantly affected by these treatments. The two ET-biosynthesis genes showed inconsistent results, with *OsACO7* slightly induced by all treatments and *OsACS1* repressed by the treatments, although none of these values were significantly different from the control plants (Fig. 6b). Transcription of the ET signaling gene *OsEIN2* showed a minor but non-significant induction following all treatments (Fig. 6c).

To obtain a more detailed understanding of the role of the ET response in biochar-induced defenses against RKNs, an *Ein2b*-RNAi line deficient in ET signaling was investigated (Fig. 6d). Confirming our earlier observations (Fig. 2a), the number of nematodes at 14 dpi was reduced in the biochar-amended Nipponbare plants. However, significant differences were not observed between the biochar-amended and non-amended plants in the *Ein2b*-RNAi line. These results imply that the ET signaling pathway is required for biochar-induced defense against *M. graminicola* in rice.

## Discussion

In this study, we investigated the effect of biochar on different stages of the infection process of the RKN *M. graminicola* in rice. All of the experiments were performed using biochar pyrolyzed from holm oak wood. The executed *in vitro* bioassays showed that the biochar exudates did not have a direct negative effect on the survival or infectivity of the RKNs.

After optimization of the concentration, our data showed that amending the SAP-medium with biochar at a concentration of 1.2 % not only reduced the number of galls formed on rice roots but also delayed the development of the nematodes in the roots. Biochar-induced resistance has been previously reported in other plants against different pathogens. Tomato plants treated with biochar pyrolyzed from citrus wood suppressed grey mold and powdery mildew caused by *Botrytis cinerea* and *Leveillula taurica* [7]. Biochar prepared from pepper plant waste suppressed three foliar diseases (*B. cinerea*, *C. acutatum*, *P. aphanis*) with different infection strategies in strawberry plants [22]. Recently, biochars pyrolyzed from eucalyptus wood chips and pepper plant wastes were found to be effective at decreasing the severity of *R. solani* infection in beans [30]. There is no standard recommended application dosage for biochar; however, the level used in this research was similar to the levels commonly reported in the literature [7, 22].

Biochar does not contain an indigenous consortium of microorganisms that can potentiate disease suppression, and the potential methods by which biochar induces systemic plant defenses against microbes has been documented in a review by Lehmann et al. [21]. The suppression of soil pathogens by biochar may stem from several mechanisms, including improved nutrient solubilization and uptake, which helps enhance plant growth and resistance to the stresses of pathogens; microbe stimulation, which promotes direct competition or parasitism against pathogens; or induced plant defense mechanisms [36]. The research presented here focused on the latter option, and to the best of our knowledge, this is the first report describing the biochemical and molecular priming mechanisms in response to biochar amendments to plants against RKN infection.

Among the biochemical responses involved in plant defense, callose deposition within the cells has been correlated to resistance in plant-pathogen interactions [37]. Callose (in the form of papillae) deposited on infections may help to reinforce the cell wall and act as a physical barrier to slow pathogen invasion [38]. In Arabidopsis, the overexpression of *RAP2.6* enhanced callose deposition in the syncytia and increased the resistance of Arabidopsis against *Heterodera schachtii* [37]. In addition, BABA application to rice was shown to induce a strong defense response that was correlated with increased callose deposition in the infected tissue [14]. However, our results showed that biochar amendments do not induce callose deposition in the root galls upon nematode inoculation. There was also no significant increase of *OsGSL1* mRNA in biochar-amended inoculated plants.

Previous research showed that the production of ET after biochar amendment had a significant impact on a range of soil and plant metabolic activities [21, 36]. Spokas et al. [35] evaluated the ET production potential from 12 different sources of biochar and observed that ET production increased (21.5 %) in non-sterile soil compared with sterile soil. This exogenous ET production from biochar-amended soil might induce resistance in plants to pathogens [21, 35]. In addition, biochar amendments have been shown to activate the SAR or ISR pathways in plants. For instance, after the application of 1–3 % biochar in the potting medium of strawberries, the relative expression of five defense-related genes (*FaPR1*, *Faolp2*, *Fraa3*, *Falox*, and *FaWRKY1*) in leaves was significantly increased. *FaPR1* and *Fraa3* are indicators of the SAR pathway, whereas *Falox* is correlated with the ISR pathway of induced resistance, indicating that biochar amendment triggered SA- and JA/ET-related gene expression in the leaves [22]. Recently, Mehari et al. [25] found that high ET sensitivity as well as SA accumulation was not required for biochar-mediated IR in tomatoes. However, JA deficiency prevented biochar-elicited IR and blocked the priming of H_2_O_2_ synthesis upon infection in tomato. The qRT-PCR analysis in the present research showed that the exogenous biochar application potentiated the increased expression of ET response genes *OsERF1* and *OsEBP89* in rice and indicated that the effect of biochar on the rice plants was dependent on ET signaling through *OsEin2B*.

Previous research from our group showed an important role of ET in the activation of JA-dependent defense against RKNs [9]. Confirming the role of ET in defense against RKNs, Fudali et al. [12] showed that ET-overproducing Arabidopsis plants are less attractive to RKNs. However, because ET is known to induce cell expansion and inhibit lignification, it was suggested that this plant growth regulator plays a major role in the development of nematode feeding sites at later time points of the infection process. As argued in the review by Kyndt et al. [39], ET most likely plays different roles at different stages of the nematode infection process, including (1) a restraining role, which occurs through the activation of nematode repellents and JA biosynthesis, and (2) an activating role, potentially through its positive effect on auxin biosynthesis, thus facilitating the radial expansion of the giant cells. The data provided by this study show that biochar-induced defenses in rice against the RKN *M. graminicola* acts at least partly through ET signaling. However, because we did not observe differences in attractiveness or giant cell development in the biochar-treated plants, further studies are required to investigate the genes and pathways that are specifically activated in the treated plants.

Our data provide evidence for biochar amendments to counteract the growth inhibition caused by nematode infection of rice plants. However, a microarray of biochar-treated Arabidopsis and lettuce indicated that the biochar-induced positive growth effects were accompanied by a down-regulation of a large suite of plant defense genes, including the JA biosynthetic pathway, defensins and most categories of secondary metabolites [40]. In contrast, our results suggest a positive effect of biochar on plant defense at the concentration of 1.2 %. Importantly, the experiments of Viger et al. [40] used higher concentrations (5 %), and it is known that an excessive activation of plant growth causes a negative effect on plant defense because of resource-limited trade-off effects.

H_2_O_2_ is an essential factor during the induction of plant defense [41]. Based on previous research, H_2_O_2_ could be effectively activated by biochar, which produces a hydroxyl radical (OH) to degrade 2-chlorobiphenyl [42]. The increased production of H_2_O_2_ in plants can lead to the polymerization of monolignols by peroxidase and the formation of lignin [33]. The results emerging from the current study demonstrate that biochar amendments induce a slight accumulation of H_2_O_2_ at the early time point, whereas subsequent nematode inoculation combined with biochar amendment results in an even stronger accumulation of H_2_O_2_ in the roots, suggesting a priming effect on the oxidative burst by biochar amendment. Similar results were reported by Taheri and Tarighi [43] in riboflavin-induced resistance in rice against *R. solani*, and enhanced H_2_O_2_ accumulation was there correlated with a higher level of lignification in riboflavin-treated inoculated plants. However, the results presented here revealed that biochar amendments did not induce enhanced lignin formation after the invasion of nematodes. These contradictory results may have been caused by the observation times used in the present research, which may have been too early to detect the accumulation of lignin.

## Conclusion

The results presented here lead us to conclude that biochar amendments in rice potting medium potentiate a primed defense reaction against the RKN *M. graminicola* and protects the plants from the negative effects of nematode infection on plant growth. The observed activation of ET responses and H_2_O_2_ accumulation may contribute to the capacity of biochar to suppress nematode infection inside roots. Clearly, additional research must be performed to quantify the extent to which biochar triggers plant defense and determine the most effective conditions to suppress nematode infection because biochar exhibits large variability in its physical and chemical properties [21]. Future research is also required to characterize the effective compounds in biochar and further identify the metabolic changes that occur in the biochar-plant-nematode interaction system.

## Methods

### Biochar and plant material

Biochar prepared from holm oak wood through pyrolysis at 650 °C for 12 to 18 h was kindly provided by PROININSO S.A. (Malaga, Spain). This biochar consists of 72.4 % dry matter (DM) (%/fresh), 77.8 % organic matter (%/DM) and 74.2 % C (%/DM) and was recently characterized and used by Vandecasteele et al. [44, 45]. The biochar was ground to a powder of <1 mm particles and stored in sealed containers until use. The biochar was added in different concentrations (0.6, 1.2, 2.5, and 5.0 %) to synthetic absorbent polymer (SAP)-substrate, which is a 1:400 (w: v) mixture of sand and a synthetic absorbent polymer [46].

Rice seeds (*Oryza sativa* cv. Nipponbare) were obtained from the US Department of Agriculture (GSOR-100). A transgenic *OsEin2b* RNAi line was kindly provided by Yinong Yang (Penn State University, State College, PA, USA). After germination at 30 °C for 4 d, the seeds were sown in polyvinyl-chloride (PVC) tubes containing SAP with or without biochar and maintained in a greenhouse at 26 °C with a 16 h/8 h light/dark regime and 70–75 % relative humidity. Each plant was fertilized twice a week with 20 ml of Hoagland solution. Two-week-old plants were used for nematode inoculation.

### Nematode culture and extraction

*M. graminicola* were maintained on *O. sativa* cv. Nipponbare in a greenhouse under the same conditions as described above. Infected roots and root galls were cut into pieces, and the nematodes were extracted using a modified flotation-sieving method [47]. The second-stage juveniles (J2s) were collected with a 25-μm sieve.

### Direct effect of biochar exudates on the behavior of nematodes

Biochar was immersed in distilled water at concentrations of 0.3, 0.6, 1.2, 2.5 and 5 % (v: v) for 1 week. The suspension was centrifuged at 12,000 g for 5 min, and the supernatant was used to test the direct toxic effect of biochar exudates on the RKNs. Approximately 200 J2s were placed into a 3.5-cm diameter well on a 6-well culture plate containing 1 ml of biochar exudates or 1 ml of distilled water for the mock treatment. After incubation for 24 h and 72 h, 1 N NaOH was dropped into the solution, and the nematodes that responded to the NaOH by changing their body shape within 3 min were considered to be alive, whereas straight nematodes that failed to respond to the NaOH were presumed to be dead [48]. The living and dead nematodes were counted under a stereomicroscope (Leica S8 APO, Leica Microsystems, Diegem, Belgium). The experiment was performed three times with 6 replicates each.

To determine the direct effect of biochar on the infectivity of nematodes, the nematodes were incubated in a biochar exudate solution for 72 h before inoculation. As a control treatment, the nematodes were incubated in water for 72 h before inoculation. Two hundred J2s were inoculated on each 2-week-old rice root. At 7 and 14 dpi, the root samples were collected and stained with acid fuchsin as described in Nahar et al. [9]. The nematodes inside the roots were counted using a stereomicroscope, and the total number of nematodes as well as the different developmental stages was counted.

### Attraction bioassays

A nematode attraction test was performed as described by Wang et al. [49]. First, 23 g of pluronic F-127 powder (Sigma Aldrich, Brussels, Belgium) was added to 100 ml of sterile water and allowed to dissolve with stirring at 4 °C for 24 h. The rice roots from 2-week-old plants were drenched with 20 ml of 1.2 % biochar exudates or water. One day later, a 1-cm-long root tip was cut and placed into a 3.5-cm well in a 6-well culture plate containing 1 ml of pluronic gel and approximately 200 J2s. The nematodes in the vicinity of the root elongation zone were counted at 9 h post-inoculation (hpi), and photographs were taken under a Leica stereomicroscope with a DFC400 camera. The experiment was performed three times with 6 replicates each.

### Resistance induced by biochar amendment

These experiments were designed to determine whether biochar amendments were effective at inducing rice defense against RKNs. Each 2-week-old rice plant maintained in SAP medium containing the appropriate concentration of biochar or control (only SAP) was inoculated with approximately 200 J2s. At 14 dpi, the root length, shoot length and fresh weight of the rice plants were measured, and the root samples were stained using the acid fuchsin method [9]. The nematodes in different developmental stages were counted under the microscope. The experiment was performed three times with 6 replicates each.

### Microscopic examination of giant cells

Microscopic examination of the giant cells was performed as described by Ji et al. [50]. Root galls were collected at 7 dpi, fixed in 1x PIPES buffer with 2 % glutaraldehyde overnight, and then dehydrated in a series of ethanol dilutions and infiltrated in Technovit 7100. The infiltrated roots were embedded in plastic cubes filled with Technovit 7100 plus Hardener II as described by the manufacturer. The embedded gall tissues were sectioned into 10-μm slices with a Leica RM2265 motorized rotary microtome (Leica Microsystems, Nussloch, Germany). Sections of the galls were maintained on cover glass and stained in 0.05 % toluidine blue for 5 min. Microscopic observations were performed using a BX 51 system microscope (Olympus Optical Company, Tokyo, Japan) at a 40x magnification, and images were obtained with an Olympus ColorView III camera. The experiment was repeated twice, and 10 galls from each treatment were observed.

### Microscopic observation of callose deposition

Callose deposition was detected according to Millet et al. [51] with minor modifications. Briefly, rice roots amended with 1.2 % biochar or untreated control plants were collected at 7 dpi. Ten root galls from each treatment were collected and fixed in a 3:1 ethanol: acetic acid solution overnight and then dehydrated in ethanol dilutions of 70, 50, and 30 % in sequence. Finally, the root galls were stained with 0.01 % aniline blue solution using vacuum infiltration. Callose deposition of the root galls was examined under UV light using a Nikon Eclipse Ti-E epifluorescence microscope (excitation, 390 nm; emission 460 nm). Quantification of the callose depositions was performed using ImageJ software.

### H_2_O_2_ and lignin quantitation

The *in planta* accumulation of H_2_O_2_ in the rice roots was determined using the trichloroacetic acid (TCA) method [52]. The roots of rice plants grown in SAP amended with 1.2 % biochar or the roots of untreated control plant roots were collected at 6, 24, and 72 hpi with RKNs. One hundred milligrams of fresh root tissue was ground in liquid nitrogen and homogenized with 0.8 ml of 0.1 % TCA. The homogenate was centrifuged, and the same volume of 10 mM potassium phosphate buffer (PPB, pH 7.0) and 1 M KI was added as the supernatant. The absorbance of the supernatant was read at 390 nm using a CLARIOstar microplate reader (BMG Labtech, Temse, Belgium). The concentration of H_2_O_2_ was estimated using a standard curve, where 0.1 μm to 1 mM H_2_O_2_ was diluted with the same ratio of TCA, PPB and KI. Each experiment was performed twice with 4 replicate samples each, and each replicate was a pool of 6 individual plants.

The accumulation of lignin was quantified according to the acetyl bromide (AcBr) method as described by Vanholme et al. [53]. The roots of rice plant grown in SAP amended with 1.2 % biochar or the roots of untreated control plant roots were collected just before inoculation (0 h) and 24 h after inoculation with the RKNs. The fresh roots were dried in a speedvac (−20 °C, 3 days) and ground in a mortar with liquid nitrogen. The ground subsamples (at least 5 mg) were subjected to sequential extractions in 2-ml plastic tubes for 30 min (each) using water (98 °C), ethanol (76 °C), chloroform (59 °C), and acetone (54 °C). The remaining cell wall residue was dried and weighed again. The absorbance of lignin was measured at 280 nm using a Nano-Drop® ND-1000 spectrophotometer (NanoDrop Technologies, Wilmington, DE, USA). The lignin concentration was calculated using the law of Bouguer-Lambert-Beer, where A = є × l × c, є = 17.75 l g^−1^ cm^−1^ and l = 0.1 cm [54]. Each experiment was performed twice with 4 replicate samples each. Each replicate was pooled from 6 individual plants.

### RNA extraction, reverse transcription and qRT-PCR

To detect the expression level of different plant defense-related genes, the roots of rice plants grown in SAP amended with 1.2 % biochar or the roots of untreated control plants were collected at 24 hpi. In each treatment, the roots of six plants were pooled and ground in liquid nitrogen. RNA extraction was performed using the NucleoSpin kit (Macherey-Nagel, Düren, Germany), which includes a DNase treatment. In total, 2 μg of RNA was used to synthesize cDNA with the SuperScript® II Reverse Transcriptase Kit (Invitrogen, Karlsrube, Germany). The primer sequences of defense-related genes and internal reference genes are listed in Table 1. All of the qRT-PCR reactions were performed in triplicate with two independent biological replicates. The qRT-PCR reactions were run on a Rotor-Gene RG-3000 machine (Corbett Life Science, Belgium) under the following conditions: 95 °C for 5 min at 1 cycle; and 95 °C for 25 s, 58 °C for 40 s, and 72 °C for 25 s for 40 cycles. The relative transcription levels were normalized using data from 3 internal reference genes, and statistical analyses were performed using the software Rest 2009 [55]. The relative expression level of each gene is shown as the fold change compared with the transcript level in the non-amended and non-inoculated control plants (set at an expression level of 1).

**Table 1:** Primers of the reference and target genes used in qRT-PCR analysis, with GenBank accession/locus numbers

Genes	GenBank accession or locus number	Primer sequences (5’ → 3’)	Function
*OsEif5C*	SM00515	F: CACGTTACGGTGACACCTTTT	Reference gene
		R: GACGCTCTCCTTCTTCCTCAG	
*OsEXP*	LOC_Os03g27010	F: TGTGAGCAGCTTCTCGTTTG	Reference gene
		R: TGTTGTTGCCTGTGAGATCG	
*OsEXPnarsai*	LOC_Os07g02340	F: AGGAACATGGAGAAGAACAAGG	Reference gene
		R: CAGAGGTGGTGCAGATGAAA	
*OsGSL1*	AP001389	F: TGAGGACCTGCCACGATT	Callose biosynthesis
		R: CACGCTGATTGCGAACAT	
*OsRbohB*	NM001049555.1	F: CTGGACAGGACCAAGAGCAG	H_2_O_2_ production
		R: ATCTTGAACGGAGCAGCACA	
*OsEBP89*	LOC_Os03g08460	F: TGACGATCTTGCTGAACTGAA	ET response
		R: CAATCCCACAAACTTTACACA	
*OsERF70*	AF193803.1	F: ACCTTGGGGGTAGCATATCG	ET response
		R: AGGGAACAGGTCCAATCACC	
*OsERF1*	LOC_Os04g46220	F: GAGTCGTCCTTCTCCTCCTC	ET response
		R: CCTCTCTTTCTCCGTTTCG	
*OsACS1*	AK071011	F: GATGGTCTCGGATGATCACA	ET biosynthesis
		R: GTCGGGGGAAAACTGAAAAT	
*OsACO7*	LOC_Os01g39860	F: GGACTACTACCAGGGCACCA	ET biosynthesis
		R: GATTAGCGCACGCGATTTTA	
*OsEIN2*	LOC_Os07g06130	F: TAGGGGGACTTTGACCATTG	ET signaling
		R: TGGAAGGGACCAGAAGTGTT	

### Statistical analysis

Except for the qRT-PCR data, which were analyzed as described above, all of the other statistical analyses were performed using SAS software version 8.0 (SAS Institute, Cary, NC). After checking for normality and homoscedasticity, significant differences among the treatments were determined according to the Duncan’s multiple range test.

## Abbreviations

RKN: Root-knot nematode
IR: Induced resistance
SAR: Systemic acquired resistance
PR: Pathogenesis-related
SA: Salicylic acid
ISR: Induced systemic resistance
PGPR: Plant growth-promoting rhizobacteria
PGPF: Plant growth-promoting fungi
ET: Ethylene
JA: Jasmonic acid
H_2_O_2_: Hydrogen peroxide
SAP: Synthetic absorbent polymer
PVC: polyvinyl chloride
DPI: Days post inoculation
TCA: Trichloroacetic acid
PPB: Potassium phosphate buffer
